# Renal Cell Cancer and Obesity

**DOI:** 10.3390/ijms23063404

**Published:** 2022-03-21

**Authors:** Anna Gluba-Brzózka, Jacek Rysz, Janusz Ławiński, Beata Franczyk

**Affiliations:** 1 Department of Nephrology, Hypertension and Family Medicine, Medical University of Lodz, 113 Żeromskiego Street, 90-549 Lodz, Poland; jacek.rysz@umed.lodz.pl (J.R.); bfranczyk-skora@wp.pl (B.F.); 2 Department of Urology, Institute of Medical Sciences, Medical College of Rzeszow University, 35-055 Rzeszow, Poland; janlaw@wp.pl

**Keywords:** renal cell cancer, obesity, hypoxia, inflammation

## Abstract

Cancers are a frequent cause of morbidity and mortality. There are many risk factors for tumours, including advanced age, personal or family history of cancer, some types of viral infections, exposure to radiation and some chemicals, smoking and alcohol consumption, as well as obesity. Increasing evidence suggest the role of obesity in the initiation and progression of various cancers, including renal cell carcinoma. Since tumours require energy for their uncontrollable growth, it appears plausible that their initiation and development is associated with the dysregulation of cells metabolism. Thus, any state characterised by an intake of excessive energy and nutrients may favour the development of various cancers. There are many factors that promote the development of renal cell carcinoma, including hypoxia, inflammation, insulin resistance, excessive adipose tissue and adipokines and others. There are also many obesity-related alterations in genes expression, including DNA methylation, single nucleotide polymorphisms, histone modification and miRNAs that can promote renal carcinogenesis. This review focuses on the impact of obesity on the risk of renal cancers development, their aggressiveness and patients’ survival.

## 1. Introduction

Cancers are the leading cause of mortality. The global prevalence of cancer, as well as cancer-related mortality rates, has been hastily increasing as a result of rising population size, ageing, and higher exposure to cancer risk factors [1,2]. According to estimations of the WHO (World Health Organization), approximately 35 million people are suffering from cancer [3]. Apart from other risk factors for cancers, such as advanced age, personal or family history of cancer, some types of viral infections, such as human papillomavirus (HPV), exposure to radiation (e.g., ultraviolet radiation from the sun) and some chemicals, smoking and alcohol consumption and also obesity have been found to be associated with the incidence of some types of cancer [4,5]. The percentage of obese individuals is increasing every year worldwide. For example, in the USA, 67.9% of the population have excessive weight [6]. The high prevalence of obesity is related to the consumption of unhealthy food in excessive amounts and the lack of physical activity, both of which results in an excessive accumulation of lipids in adipocytes and an excessive build-up of adipose tissue [7].

Large quantities of calories exceeding total energy expenditure result in the excessive accumulation of adipose tissue and triglyceride accumulation in adipocytes, especially in visceral fat tissue, leading, in consequence, to cellular hypertrophy and the promotion of inflammatory cytokine production [8,9]. Furthermore, high levels of free fatty acids (FFAs) derived from hypertrophic adipocytes can be transported to insulin-responsive organs, including the liver, pancreas, and skeletal muscle, which results in diminished insulin sensitivity [10,11]. Excessive accumulation of adipose tissue increases the susceptibility to many metabolic disorders, such as type 2 diabetes mellitus, cardiovascular diseases, and cancer. There are numerous studies confirming the role of excessive adipose tissue accumulation in the development of various disorders and diseases. The estimated life expectancy of obese patients is up to 7 years shorter compared to normal-weight individuals [12]. According to estimations, a general population-attributable fraction is 11.9% in men and 13.1% in women for all obesity-related cancers [13]. However, in the case of some cancers, the impact of obesity on the incidence of cancers is much more pronounced, e.g., oesophageal adenocarcinoma (men 29%, women 37%), gallbladder (men 11%, women 42%) and endometrium (36%) [14]. The loss of body weight appears to reduce the incidence of cancers [15,16]. It appears that renal cell carcinoma can be induced by metabolic changes resulting from numerous mutations within genes which products are involved in the regulation of metabolism, including mutations in the hypoxia pathway as well as the phosphatidylinositol-3-kinase (PI3K)/protein kinase B (Akt)/mammalian target of rapamycin (mTOR) pathway [17,18]. Lipidomic data indicate the enhanced accumulation of cholesterol and triglycerides in RCC cells. The results of some studies have indicated higher levels of cholesterol, cholesterol esters, and triglycerides in clear cell renal cell carcinoma (ccRCC) cells compared to normal tissues [19].

This review will focus on the impact of obesity on the risk of renal cancers development, their aggressiveness and patients’ survival.

## 2. Renal Cell Cancer

Renal cell cancer (RCC) represents a group of chemoresistant cancers (including clear cell RCC, papillary RCC, chromophobe RCC and renal oncocytoma) originating from the renal parenchyma, which has distinguishable histopathological subtypes, diverse molecular profiles, various clinical outcomes and treatment responses [20,21]. According to estimations, approximately 400,000 cases of new renal cell carcinomas are diagnosed every year, and the annual RCC-related mortality reaches 175,000 deaths annually [2]. RCC accounts for 85% of all kidney cancers [22]. It is considered to be the most lethal tumour of the urinary system [20]. Among various RCC tumours, ccRCC appears to be the most frequent and aggressive subtype [23]. Despite the fact that more and more cases are diagnosed at an early stage owing to the development of imaging techniques, nearly 30% of patients have locally advanced stage or distant metastasis at the time of diagnosis [24]. Most cases of RCC are sporadic; however, sometimes familial clustering is observed, especially in patients with early age of onset, with the presence of multiple and/or bilateral lesions and several malignant and benign masses within the kidneys [25,26]. RCC tumorigenesis is associated with the presence of genetic alterations, for example, the *von Hippel-Lindau* gene mutation; loss of the short arm of chromosome 3 (3p) (in most ccRCC cases); promoter hypermethylation or deletion [27,28]. Over 40% of RCC seem to be associated with obesity assessed on the basis of body mass index (BMI) but also with hypertension and cigarette smoking. Early evidence (based on quantitative analysis that included 14 studies on men and women) concerning the association between obesity and tumour risk showed that the relative risk for men and women together was 1.07 (95% CI: 1.05–1.09) per one unit of increase in BMI (corresponding to 3.1 kg for a man of an average height of 1.77 m and 2.7 kg for a woman of an average height of 1.64 m) [29]. In turn, the results of meta-analysis have demonstrated that each 5 kg/m^2^ increase in BMI may be associated with a higher risk of RCC (raised by 24% in men and 34% in women) [30].

Currently, the therapy for metastatic RCC involves molecular-targeted drugs (sorafenib, axitinib, everolimus, pazopanib) or immune checkpoint inhibitors; however, such treatment is not always effective as a result of strong side effects of these drugs as well as the individualized sensitivity of patients to the treatment [31]. Since immune-based combinations appear not to be more toxic than the use of sunitinib monotherapy, the treatment with nivolumab combined with ipilimumab, pembrolizumab plus axitinib, as well as avelumab used jointly with axitinib appears to be the new standard for the therapy of metastatic RCC [32]. The combination of ipilimumab and nivolumab was found to be well-tolerated and to increase overall survival (OS) in intermediate- and poor-risk patients (assessed on the basis of the International Metastatic RCC Database Consortium (IMRD) risk score) [32]. One of the meta-analyses demonstrated the reduction of mortality risk by 26%, associated with the use of immune-based combinations [33]. Such therapy also resulted in higher progression-free survival (PFS), complete response (CR) as well as tumour objective response rate (ORR) [33]. However, according to estimations, approximately 20–40% of patients with localized kidney cancer experience disease recurrence after curative therapy [34]. In the case of patients with distant metastasis, the 5-year survival rate is low—only 11.7% [35].

## 3. Impact of Obesity on Renal Cancers

Tumours are characterised by the uncontrollable growth of abnormal cells that possess the potential to invade or spread to the other parts of the body [3,36]. The initiation and development of cancer involve the dysregulation of cells metabolism since it requires the appropriate amount of energy as well as biosynthetic building blocks in order to maintain malignant cell proliferation [36]. Therefore, it seems that any state characterised by an intake of excessive energy and nutrients may favour the development of various cancers. Based on the review of the evidence, the World Cancer Research Fund (WCRF) provided recommendations concerning diet, nutrition, and physical activity that can significantly reduce the risk of some cancers and positively affect survival after a diagnosis [37].

The survival of cancer cells depends on the surrounding non-malignant tumour stroma cells; the presence of adipose tissue within the tumour stroma, which provides the necessary energetic reservoir, together with signalling molecules secreted by this tissue (such as adipokines, proinflammatory and proangiogenic factors), facilitates tumour progression and metastasis [38,39]. Therefore, the accumulation of excess adipose tissue and its dysfunction appear to form the optimal microenvironment for the initiation and progression of the tumour. It has been suggested that altered hormonal milieu, chronic tissue hypoxia and increased inflammatory response, cellular energetics, angiogenesis, epithelial to mesenchymal transition (EMT) and genomic instability may link the presence of obesity with the higher prevalence and onset of kidney cancers [36,40,41,42]. Increased risk of various cancers onset in obese patients may be partly associated with the enhanced secretion of endogenous hormones and steroids (e.g., sex hormones and insulin) and the subsequent disturbed balance between cell proliferation, differentiation and apoptosis [43,44]. Obesity can trigger intracellular lipid accumulation in adipocytes, insulin resistance (IR) as well as mitochondrial and endoplasmic reticulum stress [45]. The results of a large, prospective US cohort revealed that weight gain in early (18–35 years of age) and mid- (35–50 years of age) adulthood strongly correlated with the incidence of RCC, whereas weight gain after midlife (age 50 years to baseline) was not so strongly related to RCC [46]. The Metabolic Syndrome and Cancer Project (Me-Can), involving 560,388 men and women in cohorts from Norway, Austria, and Sweden, found that increased levels of BMI, blood pressure, glucose and triglycerides were associated with increased risk of RCC among men, while high BMI was most crucial in women [47]. Numerous studies have suggested that adiposity is the second most common risk factor for the initiation of cancers, including thyroid, oesophagus, liver, breast, kidney, prostate, colon and rectum [48,49,50]. White adipose tissue (WAT) is the main site of excess energy storage in the form of triacylglycerol [49]. This tissue can be transformed into metabolically active organs via cells that are contained within, such as adipocytes, immune cells (T and B lymphocytes), a stromal–vascular fraction (adipose precursor cells, endothelial cells, pericytes) as well as mast cells, macrophages, neutrophils and dendritic cells [51]. Since all these cells release numerous active biomolecules, WAT is involved in the regulation of various biological functions, both at the local and systemic levels [49]. The results of studies have indicated that adipokines, regulators of fatty acid metabolism, hormones and pro-inflammatory cytokines may stimulate the initiation and progression of obesity-related cancers [52,53].

The amount of visceral fat can also affect the aggressiveness of a tumour. According to Zhu et al. [54], a greater percentage of visceral adipose tissue is considerably correlated with a higher Fuhrman grade and could be an independent predictor of high-grade RCC in patients with stage T1a RCC. The accrual of adipose tissue is quite a well-known risk factor increasing cancer morbidity; however, the exact mechanisms responsible for this phenomenon remain elusive [55].

Apart from the increased risk of RCC, obese patients have also worse outcomes, including poorer response to traditional chemotherapy, surgery and radiation therapy [56]. However, some studies have demonstrated a positive relationship between obesity in RCC patients and considerably higher OS, cancer-specific survival (CSS) and recurrence-free survival (RFS) [57,58]. Obesity increases the risk for RCC development, but at the same time, it may decrease the risk of recurrence and increase the overall survival [59]. Parker et al. [59] revealed that overweight (BMI 25–30 kg/m^2^) and obese (BMI ≥ 30 kg/m^2^) patients more frequently had less-aggressive cancers compared with normal-weight patients (BMI < 25 kg/m^2^). Additionally, the 5-year cancer-specific survival rate was higher in overweight and obese patients (76.9%, and 81.7%, respectively) than in normal-weight individuals (62.3%). The first two groups also experienced a lower risk of RCC death. A strong relation between BMI and cancer-specific death was maintained even after the adjustment for tumour stage, size, grade, symptoms, and baseline weight loss CSS (hazard ratio (HR) = 0.47; 95% CI = 0.29–0.77) [57]. Moreover, the meta-analysis of 14 studies reported the relationship between greater BMI and markedly prolonged CSS (pooled HR = 0.59, 95% CI = 0.48–0.74) [57]. Steffens et al. [60] suggested that visceral adipose tissue may play a protective role in more advanced RCC in patients treated with sorafenib and sunitinib since patients with a larger visceral fat area (VFA) had longer progression-free survival time and OS. Moreover, a review of the records of patients who underwent nephrectomy for localized renal cell carcinoma demonstrated that overweight and obese patients with this cancer had a more favourable prognosis compared with patients with a normal BMI [61]. The results of a large clinical study of US patients with clear cell RCC treated with surgery indicated that incidence of advanced stage and advanced grade cancers in obese and overweight patients was lower compared to normal-weight patients (odds ratios of 0.61 and 0.73, respectively) [22]. This phenomenon is called the “obesity paradox”. Markedly longer survival of overweight and obese patients with kidney cancer compared with normal-weight patients is mostly observed in patients on dialysis, those with hemodynamic and metabolic disorders, including chronic kidney disease (CKD), heart failure (HF) and cardiovascular disease (CVD) [22,57,62,63]. Waalkes et al. [64] observed that in patients with organ-confined but not advanced RCC, excessive weight was associated with better survival. Additionally, another study of RCC patients reported markedly prolonged cancer-specific survival time (but not overall survival time) in those with BMI exceeding 30 kg/m^2^ who underwent radical nephrectomy. The reason why obesity increases RCC risk but, on the other hand, also improves prognosis is not yet well understood. One explanation is that obesity delays the development of sarcopenia and cachexia [45]. Another study demonstrated the presence of protective factors, including lower brain natriuretic peptide (BNP) and N-terminal pro-B-type natriuretic peptide (NTproBNP) levels as well as diminished activation of the renin-angiotensin (RAA) and sympathetic nervous system in obese patients [62]. Moreover, it has been suggested that adipocytes of obese individuals are capable of counteracting the negative effects of the catabolic state, thus enhancing their prognosis. In turn, Sanchez et al. [65] revealed that in contrast to what they expected, obese patients did not have an enhanced inflammatory state within their primary tumours. Greater inflammation and hypoxia were found in the peritumoral fat area. Based on their findings, the authors suggested that the alterations of the tumour microenvironment may be responsible for the survival advantage observed in obese RCC patients compared to normal weight ones [65]. Moreover, they demonstrated higher angiogenesis scores in obese patients. Hakimi et al. [66] indicated a better response to sunitinib in those with higher angiogenesis scores as compared to pazopanib. It has been hypothesized that despite the fact that obesity enables the formation of an environment favouring ccRCC growth via angiogenesis, it also facilitates the local delivery of TKIs (also due to enhanced angiogenesis). Another study suggested that obese RCC individuals are more likely to have the clear cell A (ccA) molecular subtype (determined on the basis of ClearCode34), which, in comparison to the clear cell B (ccB) subtype, is associated with superior OS, CSS, and RFS [67]. However, some researchers argue that the presence of the obesity paradox is the result of a bias in the study design (e.g., reverse causation bias and selection bias) and is not a real relation [49,68]. Since an increasing number of new studies have confirmed the presence of the obesity paradox, we believe that such a phenomenon exists and could be associated with the aforementioned mechanisms. Nevertheless, further studies should be performed to confirm the underlying mechanisms.

## 4. Mechanisms Linking Obesity with Cancers

### 4.1. Hypoxia

The involvement of hypoxia in RCC carcinogenesis has already been confirmed. Hyperplasia and hypertrophy of adipocytes are characteristic alterations found in obesity [45]. Unbalanced expansion of adipose tissue stimulates hypoxia, which, in turn, triggers compensatory angiogenic mechanisms in order to cope with limited supplies of oxygen and nutrients [69]. Lawler et al. [70] reported that in obese patients, adipose tissue oxygenation is lower compared to lean individuals (39.3 ± 1.5 vs. 53 ± 1.9 mmHg). Uncontrolled hypoxia has been demonstrated to facilitate tumour cell survival and propagation [71]. The hypoxic obese adipose microenvironment induces hypoxia-inducible factor 1α (HIF-1α)-related pathways and upregulates the levels of extracellular matrix (ECM) proteins (metalloproteinases; MMPs), tissue inhibitors of metalloproteinases (TIMPs) and collagens) as well as proinflammatory cytokines (e.g., tumour necrosis factor α (TNF-α), interleukin-6 (IL-6) and C-C motif chemokine ligand 2 (CCL2)). Modifications triggered by the presence of hypoxia affects the tumour itself as well as the tumour microenvironment [72]. Secreted cytokines: TNF-α, IL-6, IL-8, IL-10, and macrophage inflammatory protein 1 (MIP-1)) are involved in the promotion of angiogenesis and IR [45]. Such behaviour provides a niche for transformed infiltrating tumour cells [73,74]. Indeed, the results of studies have confirmed the involvement of proinflammatory and profibrotic factors in tumour initiation and growth in obese patients. The presence of hypoxic conditions and the flux of lipids are also associated with the intensification of immune cells infiltration and the enhanced release of inflammatory adipokines, which all lead to local and distant inflammation [75]. In obese patients, immune cells were found to change their phenotype and to promote not only inflammation but also fibrosis [76]. Such inflammatory signals promote the recruitment of myeloid cells, which are the main source of reactive oxygen species (ROS) within adipose tissue, thus aggravating the inflammatory state and leading eventually to DNA damage, genomic instability and the initiation of cancer development [77]. Obesity-associated hypoxia is a vital factor engaged in the development of insulin resistance and chronic inflammation; it also dysregulates the production of adipocytokines [78,79]. Hypoxia in adipose tissue of obese mice impairs the expression of adipocytokines, enhances adiponectin mRNA instability and upregulates the expression of CCAAT/enhancer-binding protein (C/EBP) homologous protein, which results in the reduction of adiponectin promoter activity [78].

The available data indicate the presence of hypoxia induces and stabilizes two hypoxia-inducible factors (HIFs): HIF-1α and HIF-2α, with different activities that serve as vital mediators of the cellular adaptation [71,80]. The activation of HIFs is associated with the reprogramming of cellular oxidative metabolic mechanisms, leading to bioenergetic adaptation and to decreased oxygen availability and alleviating the effects of toxic reactive oxygen species (ROS) [81]. HIF-1α was suggested to inhibit the aggressive behaviour of the tumour, while HIF-2α appeared to be the main pro-tumourigenic factor in ccRCCs. Hypoxia-inducible factor-1α (HIF-1α), which controls glycolysis and pyruvate metabolism, triggers the expression of diverse genes regulating metabolic pathways, angiogenesis, DNA replication, the synthesis of proteins, tumour metastatic potential as well as resistance to therapy [71]. HIF-1α promotes cell survival since it stimulates the expression of growth factors and hampers pro-apoptotic pathways [82]. Moreover, its actions via vascular endothelial growth factor (VEGF), VEGF receptors, cyclooxygenase-2 (COX-2), inducible nitric oxide synthase (iNOS) are associated with tumour neovascularization [83,84]. Furthermore, it was found to control cell detachment (through the downregulation of adhesion molecules) as well as to stimulate cell migration and invasion (via the actions of matrix-degrading enzymes) [71]. HIF-1α was found to stimulate tumour development via its impact on the cell cycle and apoptosis [45]. HIF-1α overexpression in RCC augmented intratumor microvessel density in xenografts [85]. Moreover, the higher expression of HIF-1α appears to be associated with a markedly worse prognosis of RCC patients compared with those with low expression [86,87]. According to studies, overexpression of HIFs in RCC is associated with the inactivation of the *von-Hippel-Lindau* (*VHL*) gene. The absence of pVHL, resulting from the inactivation of *VHL* (somatic mutations, hypermethylation), mimics the hypoxia state, which provokes a constitutive up-regulation of HIF-1α and subsequent overexpression of VEGF, platelet-derived growth factor-β (PDGF-β) and transforming growth factor β (TGF-β), which are involved in carcinogenesis and angiogenesis [88].

**Figure 1:**
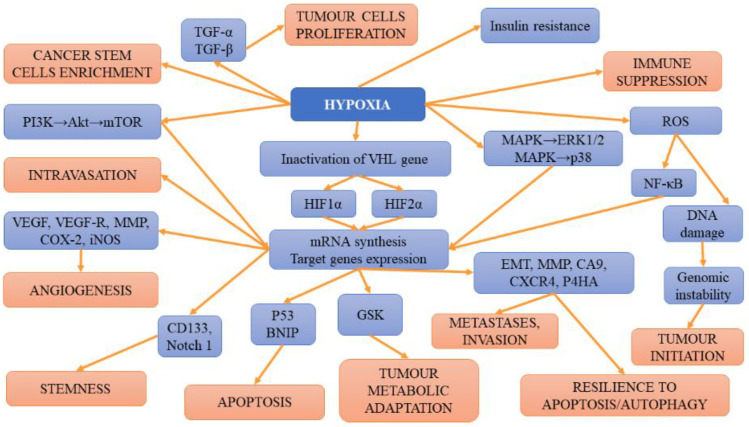
The summary of hypoxia-related mechanisms involved in tumour initiation, progression and metastases.

In turn, hypoxia related to HIF-2α has been suggested to trigger *SLC1A5* overexpression and subsequent cancer metabolic reprogramming [89]. HIF-2α regulates fatty acid metabolism [71]. The results of studies have demonstrated that renal cell cancers expressing solely HIF-2α displayed enhanced proliferation in rats compared to those with a co-expression of HIF-1α and HIF-2α [80,90]. According to some studies, in RCC expressing both factors, HIF-2α is required for the formation of ccRCC xenografts, while a HIF-1α knockdown boosts this process [91,92]. Such findings underlined the thesis that HIF-1α acts as a tumour suppressor, while HIF-2α is an oncogene. However, the results of the aforementioned studies seem to contradict this assumption. It is plausible that both factors may play various roles at different stages of tumour development. Their activity could also be affected by the presence of numerous mutations occurring in patients with ccRCC. Apart from mutations, epigenetic factors, as well as the mutual effects on each other, may also alter the presence/function of HIF-1α and HIF-2α. The results of studies analysing expression profiles support the thesis that HIF-1α may promote ccRCC at its early but also late stages of development and progression [92,93]. Gudas et al. [94] observed the correlation between greater HIF-1α expression levels and worse patient survival. However, the cumulative effect of HIF-1α and HIF-2α was not found since the co-expression of both factors did not translate into a more aggressive ccRCC phenotype [95]. However, in the mice model, the deletion of either of the factors hampered the formation of cysts and tumours triggered the presence of Vhl/Trp53 double mutation [96]. This finding confirms the pro-tumourigenic properties of both factors. The summary of hypoxia-related mechanisms involved in tumour initiation, progression and metastases is presented on Figure 1.

### 4.2. Inflammation

**Figure 2:**
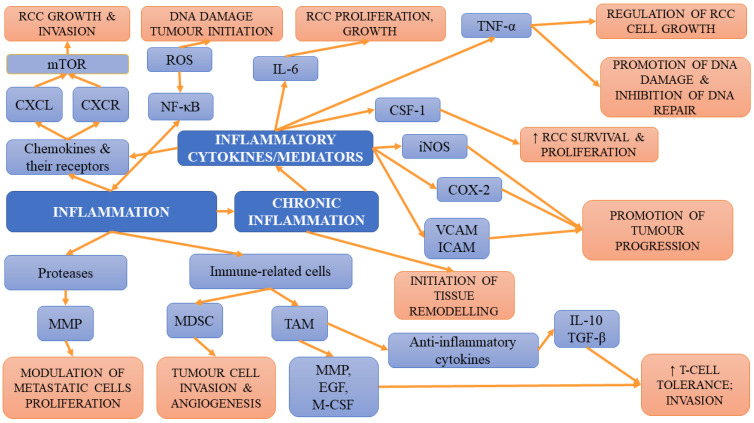
The summary of inflammation-related mechanisms involved in tumour initiation, progression and metastases.

In lean individuals, white adipose tissue containing invariant natural killer T-cells, alternatively activated macrophages, regulatory T-cells, eosinophils and T-helper type 2 cells, together with adipose cells, controls energy balance and exerts anti-inflammatory activity [97]. However, the increase in BMI is associated with adipocyte death due to hypertrophy, which results in the conversion into inflamed-WAT rich in pro-inflammatory cytokines, such as TNF-α, which subsequently affect systemic homeostasis. Such transformation favours cancer onset and progression via the TNF-α-induced release of cytokines and angiogenic factors as well as anti-apoptotic factors such as (B-cell CLL/lymphoma 2 (BCL-2)) and cyclin D1 and cyclin E in cancer cells [52,98,99]. Chronic inflammation is one of the hallmarks of tumorigenesis [100]. Chronic overload of lipids and nutrients, which is observed in obese individuals, can result in the development of an inflammatory state within adipose tissue, leading to many pathologies, including diabetes mellitus and metabolic syndrome [101,102]. However, the development of these conditions is preceded by the impairment of immunity and immune responses, which, in the long-term perspective, may favour the initiation and progression of tumours [103]. Adipose tissue is a rich source of immune cells, especially macrophages, which can, under favourable conditions, contribute to the development of cancer [76]. Adipose tissue of lean individuals is capable of releasing a variety of anti-inflammatory cells, such as T helper type 2 (Th2) T cells, alternatively activated (M2) macrophages, and regulatory T-cells [104]. However, in obese patients, the increase in the amount and dimensions of adipocytes is associated with the secretion of inflammatory factors, adipokines and cytokines (leptin, monocyte chemoattractant protein 1 (MCP-1), TNF-α, IL-6). Moreover, the activation of T helper type 1 (Th1) cells is observed. Th1 and cluster of differentiation 81 (CD81) T-cells were demonstrated to release high amounts of interferon γ (IFN-γ), leading to the worsening of inflammation within adipose tissue. Apart from the aforementioned changes, obesity also activates and upregulates intracellular pathways (signal transducer and activator of transcription 3 (STAT3), nuclear factor kappa-light-chain-enhancer of activated B-cells (NF-κB), COX-2) that are responsible for the aggravation of inflammation and cellular proliferation but also for blocking the apoptosis [105,106]. The presence of elevated concentrations of serum C-reactive protein (CRP), IL-6, TNF-α, leukocyte and higher neutrophil count in obesity-induced chronic inflammation results in the accumulation of macrophages in the adipose tissue of obese individuals [107,108,109]. The proliferation of macrophages leads to macrophage accumulation in the course of obesity development and is followed by greater migration and aggravated accumulation of macrophages in adipose tissue (mediated by the CCL2/IL-1β/C-X-C motif chemokine ligand 12 (CXCL12) signalling pathway) [110,111]. Furthermore, obesity stimulates the retention of macrophages in adipose tissue [112]. Both macrophages recruited via MCP-1, TNF-α and the increase in IFN-γ concentrations in adipose tissue contribute to the switch from the M2 pro-repair state into the activated/inflammatory (M1) macrophage phenotype associated with the enhanced expression of major histocompatibility complex (MHC) class II and pro-inflammatory cytokines [113]. The cJun NH2-terminal kinase, which is necessary for such polarization, also plays an important role in obesity-related inflammation and insulin resistance [114]. The results of studies have revealed that in obese patients, ECM displays a greater capability to polarize macrophages to the M2-like phenotype compared to ECM from lean patients [115]. According to Springer et al. [87], obesity-associated interstitial fibrosis, which stimulates a macrophage phenotype switch into macrophages similar to tumour-associated macrophages (TAM), may partly explain the link between obesity and cancers. Activated macrophages released large amounts of inflammatory cytokines, e.g., COX-2, TNF-α, IL-6 and plasminogen activator inhibitor-1 (PAI-1), aggravating the state of chronic inflammation and enhancing insulin resistance and the risk of cancer initiation [116]. Moreover, they were found to stimulate both stromal vascularisation and angiogenesis, which contribute to tumour progression [117]. TAMs that have an M2-like phenotype will promote tumour growth and hinder antitumor immune cells [118,119]. The release of cytokines by macrophages is associated with enhanced tumour growth, malignancy, ECM remodelling as well as angiogenesis. Through the release of matrix metalloproteinases and cathepsins degrading extracellular matrix proteins, TAMs also favour tumour progression and invasion [119]. The production of vascular endothelial growth factor and TGF-β by TAMs enables the angiogenesis necessary for tumour progression [120]. Not only macrophages but also adipocytes release inflammatory cytokines affecting cancer cell inflammation. Inflammation facilitates the EMT, which, in turn, enhances both the metastatic potential of tumour cells and genomic instability [105]; it was demonstrated not only to stimulate cancer cell survival and progression but also to limit adaptive immunity. Cardillo et al. [121] demonstrated that levels of interleukin-10 protein were higher in more advanced TNM stage (pT3) tumours. Moreover, they suggested that IL-6 and IL-10 and heat shock protein 90 (HSP-90) may be useful markers of the development and progression of renal-cell carcinomas. In turn, TNF-α was found not only to stimulate the proliferation and metastasis of RCC cells but also to be involved in the glycogen synthase kinase 3 beta (GSK3β)-mediated epithelial–mesenchymal transition in RCC [122]. In obesity, the enhanced formation of ROS, together with pro-inflammatory cytokines and inflammatory state mediators (NF-κB and COX-2), favours cell apoptosis, proliferation, and invasion. Cyclooxygenase-3 has been reported to be overexpressed in multiple cancers, including RCC [123]. Moreover, COX-2 and insulin-like growth factor type 1 receptor (IGF-1R) may act synergistically, enhancing the oncogenesis and progression of RCC [124]. In obese individuals, the leukocytes present within adipose tissue can also promote the process of oncogenesis through the release of numerous cytokines. The results of some studies have suggested that adipose-derived stem cells (ASCs) may act as a potential tumour promoter for different cancer cell types [125]. They can promote tumour progression and invasiveness via the activation of several intracellular signals as well as elective tumour homing capacity. Figure 2. Presents inflammation-related mechanisms involved in tumour initiation, progression and metastases.

### 4.3. Insulin Resistance

Excess caloric intake accompanied by adipocyte hypertrophic growth results in disturbed insulin sensitivity and cellular stress. The rise in glucose levels in obese individuals facilitates the formation of the pro-tumour microenvironment [106]. Insulin resistance, observed frequently in obese individuals, is associated with an early rise in insulin-like growth factor 1 (IGF-1) levels and IGF-1 receptors in WAT and other tissues [126]. Diminished synthesis of adiponectin in obese individuals is associated with the development of insulin resistance, which then results in hyperinsulinemia [127,128]. Insulin, IGF1 and TNF-α have been demonstrated to negatively affect the production of sex-hormone-binding globulin (SHBG), which is the crucial carrier of testosterone and oestradiol in the plasma, thus contributing to the rise in sex steroid bioavailability and the potential increase in the risk of tumours [129,130]. Prolonged and chronic hyperinsulinemia decreases the release of insulin-like growth factor (IGF)-binding protein-1 and 2 (IGFBP1 and 2), thus contributing to the increase in the levels of bioavailable IGF1, which, in consequence, induces cell proliferation and inhibits apoptosis, favouring the formation of tumours [43,131]. Another study demonstrated that despite being capable of stimulating cancer growth, insulin, which is one of the vital anabolic hormones, is not carcinogenic [132].

According to studies, elevated serum insulin levels prevent autophagocytosis, proteasome activity and apoptosis. Therefore, it may exert anti-apoptotic and mitogenic effects [133,134,135]. In healthy kidney cells, insulin exerts a suppressing inhibitory effect on renal gluconeogenesis (it inhibits the expression and activity of gluconeogenic enzymes) [136]. However, the situation changes in RCC cells. It was also found that the expression of IR on RCC cells was inversely associated with the cancer stage as well as the occurrence of distant metastases. In healthy persons, the expression of IR can be found in absorptive cells along the renal tubule [135]. Takahashi et al. [137] demonstrated an inverse correlation between IR expression in RCC tumours and disease progression. According to the authors, IR expression is decreased in patients with tumour stage pT2 to pT4 and those with metastatic disease. However, another study indicated that the absence of IR on RCC cells lines is not associated with the inhibition of insulin activity on RCC cells since these cells remain susceptible to insulin stimulation due to the presence of IGF-1R [135]. Moreover, Sciacca et al. [132] pointed out that IGF1 appears to be a more potent stimulator of cancer cell proliferation than insulin. Solarek et al. [135] demonstrated that IGFs and insulin may promote RCC cell viability and proliferation. IGF-1 stimulates the survival and proliferation of cancer cells through GSK3β-mediated NF-κB activation and via the blockade of cancer cells apoptosis through Rat sarcoma virus (Ras)/mitogen-activated protein kinase (MAPK) /Akt pathway-mediated inactivation of the Bcl-2 antagonist of cell death (BAD), thus preventing Bcl-2 suppression [138]. IGF-1 can promote angiogenesis not only via the HIF-1α and VEGF-C pathway but also by direct impact on vascular and lymphatic endothelial cells [49]. The expression of both IGF1 and IGF-1R within the same cancer confirms the existence of an autocrine–paracrine signalling loop of RCC cell stimulation [139]. Insulin resistance-induced alterations in the composition of the IGF family (comprising IGF-1, IGF-2, their receptors, IGF-1R and IGF-2R, and six types of IGF binding proteins (IGFBPs), IGFBP-1 to 6, have been demonstrated to be of key importance in the formation and progression of tumours. Solarek et al. [135] provided evidence that in RCC, IGF signalling is mostly associated with circulating ligand proteins—IGF1 and IGF2, obtained from sources other than RCC cells themselves. They suggested that tumour-associated endothelial cells expressing IGF may constitute a good source.

Intracellular signalling pathways associated with the stimulation of IR and IGFR1 are different. IGFR1 signalling is related to the regulation of genes participating in proliferation and subsequent mitogenic activity and the control of cancer cell motility, adhesion and angiogenesis [139,140]. The evidence for the importance of IGF1 signalling in cancers was obtained in a study showing that IGF-1 binding to IGF-1R and the downregulation of this receptor are similarly efficient in the inhibition of RCC cell lines’ growth [141,142]. Rasmuson et al. [143] demonstrated that high serum IGF-1 levels at diagnosis correlated with better prognosis in RCC. Other studies revealed that IGF-1 that is bound to IGF-1R may stimulate mitosis and cell migration, prevent apoptosis (via the activation of MAPK and PI3K signalling pathways) as well as enhance tumour angiogenesis via raising vascular endothelial growth factor levels [144,145]. The activity of IGF-1 can be modulated by the competitive binding of IGFBP-3. Microarray analysis showed increased *IGFBP-3* mRNA in 63% of clear cell renal cell carcinomas and the higher IGFBP-3 staining intensity in high grade (Fuhrman grades 3 and 4) clear cell renal cell carcinomas [146]. The Cremona study at the 15th year of follow-up revealed that patients in the group with the highest quintile of serum insulin had a 62% higher risk of cancer mortality [147].

### 4.4. Adipokines and Adipose Tissue

Adipose tissue is not only a storage place for lipids, but it also acts as active endocrine tissue, secreting numerous adipokines, including adiponectin, leptin, resistin, PAI 1, TNF-α, VEGF, and IL-6 [127,128,148]. The results of studies point to adipocytes as strong candidates facilitating the carcinogenesis process and also modulating the tumour microenvironment [149]. The crosstalk between adipocytes and cancer cells have been found to induce morphological and functional alterations, including the delipidation of adipocytes, enhanced release of proinflammatory molecules (PAI-1, IL-6 and IL-8) and reduction in adipocyte terminal differentiation markers; therefore, cancer-related adipocytes gain a fibroblast-resembling and cancer-promoting phenotypes [150,151]. Zhang et al. [152] observed in an animal model of obesity and cancer a 6-fold increase of adipose stromal cells in the systemic circulation, which contributed to an increase in tumour vascularisation and the enhanced proliferation of neighbouring cancerous cells. Adipose stromal cells were also found to stimulate tumour metastasis.

#### 4.4.1. Leptin

Leptin is an adipocyte-specific protein synthesized primarily by white adipose tissue; it regulates satiety and body weight [153]. Abnormal levels and/or the dysfunction of leptin are associated with excessive weight and uncontrolled energy intake. The analysis of leptin concentration in obese and normal-weight individuals revealed 5- to 10-fold greater levels in the first group [154]. Moreover, the relationship between higher leptin and the risk of metabolic diseases, including cancer, has been revealed. The results of studies have suggested that leptin could be the link between obesity and cancer. Leptin signalling via the leptin receptor (LEPR) was found to be associated with RCC invasion [155,156,157]. Leptin, as a multifunctional hormone, is involved in the regulation of energy expenditure, the inhibition of apoptosis and the stimulation of proliferation and angiogenesis [158,159,160]. The results of studies have indicated the association between increased serum leptin concentrations and the overexpression of leptin receptors and RCC invasion and progression [158,159,160]. The levels of leptin have been reported to correlate with greater adiposity in humans, as well as with a higher prevalence of cancers in obese individuals. This molecule enhances pro-inflammatory signalling within the cell, stimulates mitogenic effects, promotes angiogenesis and induces EMT, thus contributing to tumour progression [161,162]. Leptin was found to activate MAPK, Jak/Stat, and PI3K/AKT pathways, thus promoting oncogenic signalling, angiogenesis, and immunomodulation, leading to the enhanced proliferation and survival of cancer cells [49]. It was suggested that the activation of the extracellular signal-regulated kinases (ERK1/2) and Janus kinase/signal transducer and activator of transcription 3 (JAK/STAT3) signalling pathways were involved in the leptin-mediated proliferation of RCC in Caki-2 cells [155,163]. Leptin-boosted carcinogenesis is associated with the stimulation of cell proliferation, inhibition of apoptosis, and the upregulation of VEGF via HIF-1α and NF-κB [159]. In obese patients, higher leptin levels were found to promote the proliferation of cancer cells and metastasis [164].

#### 4.4.2. Adiponectin

Adiponectin, which exerts insulin-sensitizing, anti-inflammatory and anti-apoptotic properties, regulates many crucial processes such as glucose and lipid metabolism [165]. Adiponectin, mainly secreted by white adipose tissue, is a regulator of glucose and lipid metabolism and energy homeostasis. Obesity has been found to decrease serum adiponectin, while weight loss may raise serum adiponectin levels [166]. Serum adiponectin was found to be adversely related to RCC [167]. Adiponectin seems to have antitumor properties since it hampers angiogenesis and reduces macrophage infiltration via the suppressing of mTOR and Stat3 pathways and the stimulation of 5’AMP-activated protein kinase (AMPK) and caspase activity [39,168]. It hampers tumour cell growth and proliferation via the impairment of intracellular mediators of, e.g., PI3K-AKT, ERK1 and ERK2, STAT3 and WNT-β-catenin signalling, leading to greater cell cycle arrest and apoptosis [169]. Thus, it appears to protect against carcinogenesis [170]. This suggestion was confirmed in an animal study in which mice with hypoadiponectinemia suffered from accelerated tumour formation in the liver [171]. In another study, low blood adiponectin levels were significantly correlated with tumour size and metastasis of RCC [172]. Considerably lower serum levels of total and high molecular weight (HMW) adiponectin were reported in RCC patients with metastasis compared to non-metastatic RCC [173]. Moreover, de Martino et al. [174] observed that lower preoperative serum adiponectin is associated with features of biologically aggressive RCC, metastasis, and survival. However, Horiguchi et al. [157] found an inverse correlation between serum total adiponectin level and BMI as well as between high concentrations of total adiponectin levels and RCC aggressiveness and poor survival. Kelesidis et al. [175] found that adiponectin signalling could be partially enhanced by T-cadherin, which also strongly binds HMW adiponectin. Several studies confirmed the role of T-cadherin in the regulation of the progression of several types of cancers via the impact on tumour cell proliferation and migration and intratumoral angiogenesis [176]. Ito et al. [177] suggested that while adiponectin released from perinephric adipose tissue may impact RCC aggressiveness via the alteration of the tumour microenvironment, the levels of adiponectin in perinephric fat-conditioned medium seem not to be considerably related to RCC aggressiveness [177]. Moreover, exogenous adiponectin was shown to boost cancer cell proliferation in vitro in a mechanism associated with the inhibition of apoptosis and modulated by protein kinase and apoptosis-related protein activity. The discrepancies in the results of studies are not clear; however, they could be explained by the fact that the presence, for example, of diabetes, hypertension, and cardiovascular disease, but also racial background and circadian rhythmicity, may affect the concentrations of circulating adiponectins [178,179]. Moreover, Grossmann et al. [180] suggested that the levels of adiponectin and leptin and also the balance between them appear to be critical factors in obesity-related carcinogenesis.

### 4.5. Fatty Acids

There are many reports concerning an enhanced efflux of fatty acids in obese individuals. Tumour aggressiveness relies on the increase in lipid usage within cancer cells, and this is obtained via the synthesis of fatty acids (FA) de novo [181]. Fatty acids enable cells to raise energetic yields, while fatty acids derivatives are considered to be key components of tumour cell structure [182]. While endogenous lipogenesis becomes insufficient in rapidly growing, aggressive cancers, they increase the uptake of fatty acids from the outside [183]. At that time, cancer-associated adipocytes launch the hydrolysis of triglycerides to release FA. Fatty acid-binding protein 4 (FABP4) was found to facilitate the transfer of adipocyte-derived FAs between cancer-associated adipocytes and cancer cells, while CD36 facilitates FA uptake, thus providing tumours with sufficient energy to grow and progress [184,185].

The analysis of a database of more than 2000 ccRCC patients who underwent renal mass surgery revealed considerably upregulated fatty acid synthase (FASN) in the group with normal BMI, which was downregulated in obese patients [22]. Moreover, higher FASN expression was correlated with the presence of more aggressive disease and poor prognosis in several cancer types, including RCC [186,187,188]. Overexpression of FASN was suggested to decrease cancer-specific survival [186]. FASN was found to be associated with poor prognosis in RCC and other cancers [186]. Furthermore, an in vitro study demonstrated that the pharmacological inhibition of FASN hampered RCC tumour growth [189]. However, Albiges et al. [58] observed marked downregulation of *FASN* gene expression in obese RCC patients compared to individuals with normal BMI (*p* = 0.034). Obese patients with high FASN-expression had significantly longer OS (36.8 months–median), while patients with normal BMI had a mean survival of only about 15 months (median) (*p* = 0.002).

**Figure 3:**
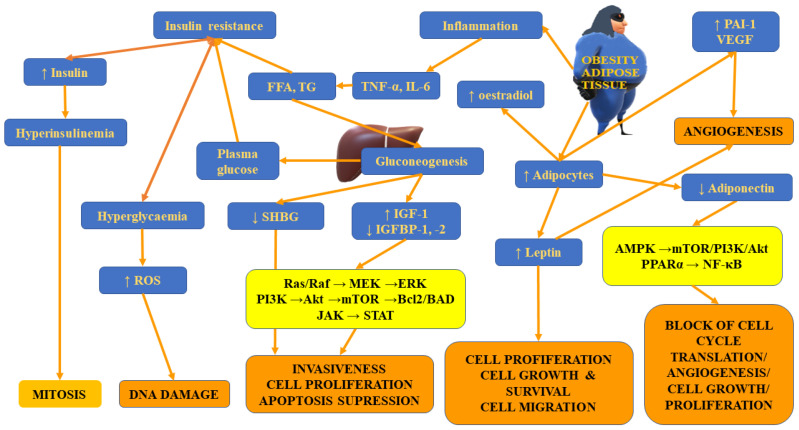
The summary of insulin-resistance- and adipose-tissue-related mechanisms involved in tumour initiation, progression and metastases.

Apart from differences in *FASN* expression, Hakimi et al. [22] observed distinct expression of the immediate upstream enzyme acetyl-CoA carboxylase (*ACACA*) gene and the level of the encoded protein—acetyl-CoA carboxylase (ACC) between patients with normal weight and obese ones. Both FASN and ACC are rate-limiting enzymes participating in the regulation of the biosynthesis and metabolism of fatty acids. This process was found to be essential for tumour growth [190]. Figure 3 presents insulin-resistance- and adipose-tissue-related mechanisms involved in tumour initiation, progression and metastases.

### 4.6. Peroxisome Proliferator-Activated Receptors (PPARs)

The PPARs superfamily (PPARα, PPARβ, and PPARγ) of ligand-activated transcriptional factors belonging to nuclear hormone receptors exerts diverse physiological functions. They have been suggested to play a role in both adipocyte differentiation and tumorigenesis [45]. Only a few studies have reported the pro-carcinogenic effects of PPARα. Yaghoubizadeh et al. [191] demonstrated that the overexpression of PPARα in the tumour microenvironment (TME) was associated with a worse prognosis. In turn, PPARγ was found to regulate adipocyte differentiation, improve IR and be involved in the development of inflammation, autoimmune diseases, and cancers. High expression of PPARγ was observed in RCC tissue, while PPARγ agonists (pioglitazone and troglitazone) and the endogenous ligand (15-deoxy-Delta12,14-prostaglandin J(2) (15dPGJ(2)) inhibited human RCC cell proliferation via the stimulation of apoptosis and G0/G1cell cycle arrest [192,193]. Apart from activation of apoptosis, 15-deoxy-delta12,14-prostaglandin J2 was found to exert cytotoxic effects on RCC cells through the stimulation of c-Jun N terminal kinase (JNK)/MAPK and Akt pathways [194]. Deguchi et al. [195] found that overexpression of PPARβ/δ was associated with the higher activation of β-catenin as well as connexin 43, eukaryotic translation initiation factor 4 gamma 1 (EIF4G1), platelet-derived growth factor receptor beta (PDGFRβ), Akt1 and cyclin-dependent kinase 1 (CDK1), which stimulated tumour (colorectal) progression. Apart from these factors, PPARβ/δ also triggered IL-6/STAT3-mediated inflammation and promoted the expression of several pro-metastatic genes [196,197]. However, another study indicated that PPARγ-mediated upregulation of phosphatase and tensin homolog (PTEN) resulted in the inhibition of PI3K signalling, thus reducing the self-renewal and aggressiveness of cancer stem cells [191,198,199]. PPARγ was also revealed to exert pro-apoptotic and anti-inflammatory properties as well as to decrease ECM remodelling and EMT, thus limiting tumour metastasis [200,201,202]. The aforementioned data show that there is no consensus concerning the role of PPARs in cancer development due to their dual role in this process.

### 4.7. DNA Hypermethylation, miRNAs and Single Nucleotide Polymorphisms (SNPs)

According to studies, in cancers, there are many mechanisms affecting obesity-related gene expression, including DNA methylation, SNPs, histone modification and miRNAs [203]. The analysis of the impact of hypermethylation of 20 genes related to obesity on renal carcinogenesis and prognosis revealed that genes of neuropeptide Y, leptin, and leptin receptor were considerably more hypermethylated compared to normal adjacent parenchyma tissue (*p* < 0.0001) [203]. The hypermethylation of the leptin receptor gene was demonstrated to decrease the expression of the encoded protein, and some researchers have suggested that this can enhance the risk of disease recurrence. The reduction in receptor levels may translate into a decreased ability to exert antimetastatic effects mediated by the activation of matrix metalloproteinase enzymes. In turn, the examination of the promoter methylation status of 10 biologically significant tumour suppressor and cancer genes (*VHL*, *p16(INK4a)*, *p14(ARF)*, *APC*, *MGMT*, *GSTP1*, *RARbeta2*, *RASSF1A*, *E-cadherin*, and *Timp-3*) in 100 kidney tumours demonstrated the occurrence of hypermethylation in all of the histological cell types and grades and stages [204]. This process is common in cancers and may occur relatively early in their development, potentially disrupting critical pathways and favouring kidney tumorigenesis. The profile of hypermethylation can be used to diagnose the type of kidney cancer and predict the patient’s clinical outcome [204]. High-resolution epigenomic and genomic maps of RCC tumours have demonstrated that RCCs are characterized by a considerably higher number of hypermethylated loci and that most of the differentially methylated regions were localized in the enhancer regions of the kidney genome [205]. Despite the identification of numerous hypermethylated loci in RCC, there are hardly any reports describing their association with clinical outcomes and disease-free survival in RCC [203]. In the longer perspective, the determination of aberrations in DNA methylation may help to clearly distinguish RCC from normal tissues.

Apart from the methylation, the presence of Gln223Arg (A/G) rs1137101 SNP was also found to affect the risk of RCC and patients’ survival [206]. The GG genotype is associated with more aggressive tumour behaviour and shorter survival compared with GA and AA genotypes. Other studies have suggested the relationship between RCC risk and polymorphisms in obesity-related genes, such as *FTO* and *ADIPOQ*, and genes in the mTOR signalling pathways [207,208,209]. Brennan et al. [209] observed that the *FTO* A allele, which is associated with increased BMI, is also correlated with a weak increased risk of kidney cancer, which is more apparent before the age of 50 (OR = 1.44, CI 1.09–1.90). In turn, Zhang et al. [208] found significantly higher ccRCC risk in carriers of the rs182052 variant A allele of the *adiponectin* gene (adjusted OR, 1.36 and 95% CI, 1.07–1.74 for AA vs. GG, *p* = 0.013; adjusted OR, 1.27 and 95% CI, 1.04–1.56 for AA vs. GG+AG, *p* = 0.019), and this positive relationship was more evident in overweight subjects. Both ccRCC patients and healthy control subjects possessing A alleles of rs182052 had lower fasting serum adiponectin.

The analysis of genes with expressions that correlated with both ccRCC and obesity revealed five candidates: immunoglobulin heavy constant alpha 1 (*IGHA1*) and immunoglobulin κ constant (*IGKC*), which act as oncogenes, as well as monoamine oxidase A (*MAOA*), mucin-20 (*MUC20*) and transient receptor potential melastatin 3 (*TRPM3*), being tumour suppressor genes. In turn, a multiphase study of three independent genome-wide scans (of 3530 cases and 5714 controls) assessing genetic variations in obesity-related genes and RCC risk identified five RCC susceptibility loci: *IL1RAPL2* (rs10521506-G), *PLIN2* (rs2229536-A), *SMAD3* (rs4601989-A), *MED13L* (rs10850596-A) and *TSC1* (rs3761840-G) [210]. *IL1RAPL2* belongs to the interleukin-1 receptor (IL-1R) family, *PLIN2* encodes perilipin 2 (also called adipophilin or adipose differentiation-related protein), SMAD3 plays an important role in regulating glucose energy homeostasis, and TSC1 is a critical tumour suppressor in mTOR pathway. Urinary perilipin 2 has been suggested as an early screening biomarker for RCC, which enables the differentiation between cancer patients and healthy controls, benign kidney tumours, noncancerous kidney diseases, and other cancers, including bladder and prostate cancers [211,212]. Comprehensive pathway analysis identified new ccRCC pathogenic factors: aryl-hydrocarbon receptor (AHR), grainyhead-like-2 (GRHL2), and KIAA0101 [213]. The expression of *GRHL2* was associated with a higher risk of disease relapse and remained statistically significant following the adjustment for grade and stage (hazard ratio (HR), 3.47, *p* = 0.012). In turn, patients with *KIAA0101*-positive expression had worse disease-free survival (HR, 3.64, *p* < 0.001). Moreover, the authors observed that the silencing of KIAA0101 was associated with a reduction in kidney cancer cell migration and invasion in vitro [213]. Many articles show a significant relationship between *LEPR* expression level and tumour aggressiveness, invasion, metastasis and clinical outcome in RCC [214,215]. Reduced LEPR levels were suggested to be associated with more aggressive tumours [216,217]. It seems that the downregulation of *LEPR* expression in RCC tumours may be related to the methylation in the promoter-associated CpG sites of LEPR [218].

Some studies have underlined the importance of selected miRNA related to obesity for the modulation of carcinogenesis [219]. For example, miR-143 overexpression was demonstrated to prevent tumour growth via inhibition of Bcl2, extracellular signal-regulated kinase-5 (ERK5) activities and KRAS (Kirsten rat sarcoma virus) oncogene [220,221,222]. The expression of oncogenic miR-221 was positively correlated with BMI (especially in women), but it was also found to regulate the pool of cancer stem cells and stimulate epithelial-to-mesenchymal transition, thus facilitating cancer tumorigenicity [164,223,224]. According to studies, miR-204-5p and miR-139-5p are important factors involved in the pathogenesis of ccRCC [213]. miR-204-5p was found to modulate ccRCC tumorigenesis and recurrence and to inversely correlate with 13 obesity-related genes [225]. Patients with a low expression of miR-204, accompanied by increased levels of miR-21, were found to have the worst prognosis compared with other groups [226]. Decreased levels of miR-204-5p were found in patients with RCC patients who progressed to metastatic disease compared with those without progression; therefore, it has been suggested that this miRNA may act as a tumour suppressor [225]. This thesis was supported by evidence obtained from in vitro studies demonstrating that the overexpression of miR-204-5p significantly limited cell migration and invasion in different cell lines [227,228,229]. In mesenchymal stem cell lines, enhanced expression of miR-204-5p stimulated adipocyte differentiation and boosted lipid droplet accumulation [3,230]. Since miR-204-5p expression was found to be positively correlated with BMI, it seems plausible that this miRNA may contribute to ccRCC recurrence via its link with obesity [225]. Other studies have reported that the tumour suppressive function of miR-139-5p is related to recurrence or metastasis. Diminished expression of miR-139-5p was observed in nephrectomised ccRCC patients with recurrence [231]. Some studies have reported that miR-139-5p levels were correlated with the survival of ccRCC patients [232].

### 4.8. Conclusions

The aforementioned data indicate the plausible association between obesity and the development of cancers. Various mechanisms can be involved in this interplay. However, at the same time, obesity appears to prolong overall survival as a result of a phenomenon called the “obesity paradox”. Therefore, it seems that this field warrants further extensive investigation. Currently, the choice of therapy in metastatic RCC patients is not based on obesity or non-obesity status since there are no unequivocal data confirming the utility of obesity or other clinical features, such as age, gender, and ethnicity, as predictive biomarkers of response to treatment. Researchers are working on the development of therapies targeting adipocytes, adipose stromal cells, and adipose endothelium; however, due to the fact that such treatments are still not tested well, especially in clinical trials, their usefulness in the treatment of RCC patients cannot be predicted. Despite the advancement in our knowledge on mechanisms involved in RCC initiation and development, there are still numerous gaps that need to be filled. We still do not fully understand the causes and pathogenesis of various tumours. Additionally, it is very important to recognise the heterogeneity of cancer cells and the consequences of their interplay. First of all, efforts should be made to establish biomarkers that would enable early detection, as well as predictive markers and those determining the response to the given treatment. Moreover, vast studies aiming at the identification of early lesions in order to improve risk stratification, cancer prognosis and control should be performed. The lack of screening tests is associated with the diagnosis of cancers in their advanced stages, which is associated with limited therapeutical options and significantly decreased chances of patients’ survival. Further studies should improve our understanding of the molecular mechanisms involved in the earliest stages of cancers and the cellular and physical properties of the tumour microenvironment that could promote the shift from premalignant to invasive disease state [233]. The unravelling of mechanisms involved in cancer progression and metastasis would enable the development of better therapies as well as solutions on how to overcome the problem of drug resistance. Finally, since studies sometimes provide conflicting results, the future challenge would involve the development of reproducible systems, allowing for the obtaining of sound evidence.

## Data Availability

Not applicable.
